# Drilling Performance of Novel Lightweight Carbon Specialty Graphites with Different Grain Sizes

**DOI:** 10.1021/acsomega.6c01467

**Published:** 2026-04-22

**Authors:** Mete Kayihan, Mustafa Yildiz, Ali Taner Kuzu, Mustafa Bakkal

**Affiliations:** † Istanbul Technical University, Mechanical Engineering Faculty, Istanbul 34467, Turkiye; ‡ 111323 TUBITAK SAGE, Ankara 06105, Turkiye; § 52967 Isik University, Engineering Faculty, Istanbul 34980, Turkiye

## Abstract

Because of their excellent machinability, low density, and great thermal stability, specialty graphite materials are being utilized more and more in precision engineering applications. The impact of the graphite particle size on drilling performance under various cutting conditions, however, has not been well studied. The drilling performance of three specialty graphite materials with various grain sizes was examined in this study. While MSG30 and MSG46 have micrometer-sized grains with greater mechanical strength, the graphite substance labeled MSG215 has a coarse grain structure (0.9 mm). High speed steel and tungsten carbide drills were used in drilling experiments at three distinct feed rates and cutting speeds. Using Taguchi experimental design and analysis of variance, the effects of cutting settings and particle size on the thrust force were examined. Workpiece temperatures were monitored during drilling using embedded thermocouples, and the correlation between the temperature evolution at four distinct depth points and cutting parameters was assessed. The dimensional accuracy, tool wear, and surface roughness were additionally evaluated. The findings show that the drilling performance is significantly impacted by graphite grain size. In comparison to fine-grained materials, coarse-grained graphite generated thrust forces that were about 30% lower and surface roughness levels that were about 20% greater. The temperature increase during drilling was minimal, owing to the strong thermal conductivity of graphite, resulting in an average workpiece temperature of 35 °C and a rise of about 15 °C under dry cutting conditions. Tool wear and thermal loads were also reduced compared with those commonly seen in conventional engineering materials. In addition to offering useful advice for choosing appropriate cutting parameters and tool materials in precision graphite machining applications, the findings provide light on the microstructure-dependent drilling behavior of specialty graphite.

## Graphical abstract

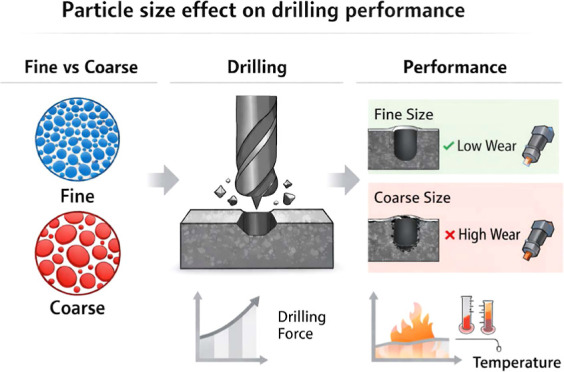

## Introduction

Graphite is a widely preferred carbon material in the industry due to its physical properties, such as high-temperature resistance, excellent electrical and thermal conductivity, chemical inertness, and low coefficient of friction. Furthermore, its lightweight and high machinability allow graphite to be used in the aviation, automotive, and nuclear industries.^1^

Besides, specialty graphites are specially processed graphite materials that offer high purity, enhanced mechanical properties, self-lubricating properties, and superior thermal/electrical performance.^2,3^ Unlike conventional graphite, they are produced through controlled manufacturing processes (e.g., isostatic pressing, CVD coating) and are used in high-technology applications. It has very low ash content (<50 ppm), which is particularly important in the semiconductor, tribology, and nuclear industries. It offers 3–5 times higher compressive and flexural strength than traditional graphite, high current-carrying capacity, and high efficiency in electrodes and batteries.^4–6^

Although graphite is frequently used in industry for machining, research on the machinability of novel specialty (fine or extremely fine grain-sized) graphite materials is quite limited. The tool wear during machining of graphite material with a diamond-coated tool is a challenging situation because of reduction.^7^ Due to the intense tool wear, they were unable to explain the wear mechanisms. Also, the machining of graphite material sometimes shows properties similar to those of the processing of compacted graphite iron. They attributed the fluctuation of the forces to the brittleness of the material and the nonuniformity of the graphite structure.^8,9^

Moreover, making machining easier and causing less tool wear with dry conditions, some work was also done on processing graphite material with micro-, nano-, and CVD-coated tools.^10–15^ Also, some micro-milling studies have been done with three different types of end mills with different parameters on the molded isostatic graphite grade. The feed parameter had the highest effect on surface roughness, which is that higher feed rates resulted in higher roughness values. Better surface quality was achieved with the diamond-coated tool compared to the other tools.^16–18^ In addition, graphite has been used with some other materials to obtain composite structures, and hole drilling/machining or mechanical property studies have been conducted for these innovative materials.^19–22^ Graphite has been obtained from graphene, a carbon allotrope, and tests have been conducted on its chemical milling studies.^23^ Graphite production is also gaining importance in this process.^24^ Although it is easily machined, whether the graphite material leaves residual stress after processing is important for the nuclear industry, which is a critical sector. Therefore, the effect of residual stress after machining has also been investigated.^25^ Studies on carbon and carbon fiber materials, similar to those on graphite, an allotrope of carbon, are quite common. These studies take both force and temperature measurements.^26,27^ While both studies target aerospace materials and lightweight automotive components, their temperature measurement strategies and cutting parameters are taken as examples.^27,28^

Despite all this work, current knowledge on the machining of carbon specialty graphites (fine and extremely fine grain-sized) remains limited; however, the lithium-ion battery technology, refractory materials, and nuclear industries, where damage must be avoided, continue to carefully monitor this material, different cutting tools such as high speed steel (HSS) and WC-Co drill bits, and their machinability. Specifically, the forces generated during the hole drilling process, which is a sample preparation process, dimensional accuracy, and most importantly temperature measurement, are a desired topic of study. Some end users in the industry are particularly interested in understanding the machining forces and temperatures of specialty graphites, which have advanced mechanical properties. The aim of this study is to shed light on hole drilling operations for these engineering materials. Following this work, comprehensive studies have been conducted on hole drilling in light carbon special graphites, focusing on potential problems that may arise in the machining of these carbon materials. Optimum cutting parameters have been selected by some studies about the graphite material for limiting the cutting force for all three-specialty graphite materials, and the tool wear of graphite has been determined. Temperature measurements were also followed for three different specialty graphite materials in some of the tests.

To the best of the authors’ knowledge, no study has systematically compared the drilling of specialty graphites with different grain sizes under controlled processes. This study addressed this gap by providing a comparative analysis of three different grain-sized specialty graphites under identical drilling conditions. Hence, the grain size is quantitively linked to thrust force, temperature, dimensional accuracy, and tool wear mechanisms. Moreover, the results statistically established parameter–effect relationships using the Taguchi design and analysis of variance (ANOVA) to determine the dominant factors on drilling performance. These findings contribute to the main understanding of grain size-dependent drilling performance in the specialty graphite industry and provide guidance for tool material selection and process optimization for suppliers and end users.

## Materials and Methods

Three different graphite materials (MSG215, MSG30, and MSG46) were used as workpieces in the study. The mechanical properties of these three different graphite materials are listed in Table 1. MSG215 was produced by the extrusion method and has an elevated grain size, which is 1 mm. The production of the rest of MSG215 involved the use of the cold isostatic pressing method, resulting in a fine-grained structure with a 0.1 and 0.09 mm grain size. One of the primary microstructural factors influencing graphite materials’ machinability is grain size. Grain size variations affect chip formation mechanisms, fracture behavior, and mechanical strength during machining. In general, fine-grained graphites are stronger and have more consistent structures, which can result in larger cutting forces but better surface quality. Coarse-grained graphites, on the other hand, are more likely to fracture, which can lead to lower cutting forces but possibly rougher surfaces. Therefore, optimizing machining parameters in specialty graphite materials requires an understanding of how grain size affects drilling performance.^1,5^

**Table 1:** Mechanical Properties of Materials

properties	MSG215	MSG30	MSG46
density (g/cm^3^)	1.74	1.85	1.9
tensile strength (MPa)	110	320	420
hardness (shore)	31	58	70
maximum grain size	1	0.1	0.09

The dimensions of the workpiece used in the study were 110 mm × 160 mm × 80 mm. Tests were conducted on a Spinner MVC 1000 vertical machining center. An intermediate plate was fabricated to secure the workpiece to the machine and force-measuring dynamometer. Force measurements were performed with a Kistler 9272 dynamometer. Data were transferred to a computer by using an amplifier from the dynamometer. These data were analyzed using CutPro software, and force–time graphs were obtained. The hole drilling tests were carried out with a three-factor and three-level L27 Taguchi experimental design. The L27 orthogonal array corresponds to the full factorial design for three factors at the three levels. Each test was repeated three times. This aimed to provide detailed observations of the effects of force results on all three phenomena: particle size, cutting speed, and feed rate. This would allow us to identify the most effective parameters and optimize the cutting parameters. Cutting parameters were determined based on in-depth literature reviews. The test equipment is shown in Figure 1.

**Figure 1:**
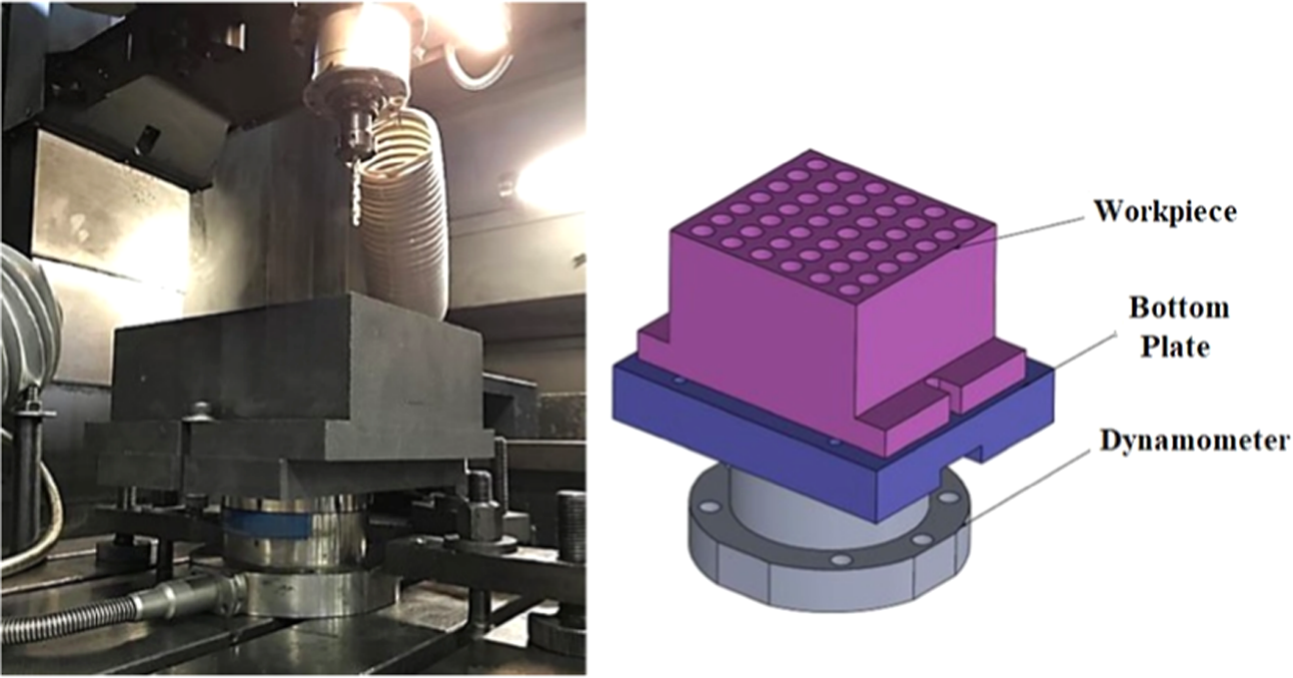
Test equipment.

Two different drills were used in the study: High speed steel (HSS) and tungsten carbide (WC). The HSS drill set is from Stock, and the WC drill set is from Ceratizit brand. The helix angle and tip angle of the HSS tool are 37° and 108°, respectively. The tool diameter of the HSS tool is 11.982 mm. The helix angle and tip angle of the WC-Co tool are 30° and 118°, respectively. The tool diameter of the HSS tool is 11.998 mm. The selected drilling speed and feed rates were determined based on the thermal and wear resistance characteristics of the drill bits, as well as recommendations reported in the literature for graphite machining. Generally, lower cutting speeds (50–100 m/min) were used for HSS drills to limit thermal softening and excessive wear, whereas higher speeds (100–200 m/min) were selected for WC-Co drills due to their superior hot hardness and wear resistance.^16,17^ In the study, drilling operations were performed with HSS and WC drills at different cutting parameters, and these parameters are summarized in Table 2.

**Table 2:** Test Parameters

HSS cutting tool	WC cutting tool
cutting speed (50/75/100—m/min)	cutting speed (100/150/200—m/min)
feed rate (0.15/0.2/0.25—mm/rev)	feed rate (0.15/0.2/0.25—mm/rev)

For the measurement of temperature during drilling, 1 mm Ø holes were drilled using a HIOKI data acquisition device with thermocouples positioned 0.2 mm closer to the cylindrical sample area. The hole diameter was kept minimal (1 mm) to avoid disturbing the local heat flow. Data from these four points were collected by using OMEGA K-type thermocouples. Because no literature was available on this subject, using K-type thermocouples was deemed to be more appropriate. The response time of the thermocouple is 0.003 s, which is sufficiently short relative to the drilling duration, ensuring reliable capture of transient temperature variations.

The four thermocouples were positioned, respectively, at a depth of 9 mm on the surface from the hole center to capture subsurface temperature evolution in the heat-affected zone, which is presented in Figure 3. This location was selected to avoid direct tool–sensor interference while ensuring sensitivity to heat generated at the cutting interface. The thermocouple was calibrated using a controlled boiling of water, and this was confirmed at multiple reference temperatures. The thermocouple was fixed using high-temperature thermal paste to ensure stable positioning and good thermal contact during drilling.

**Figure 2:**
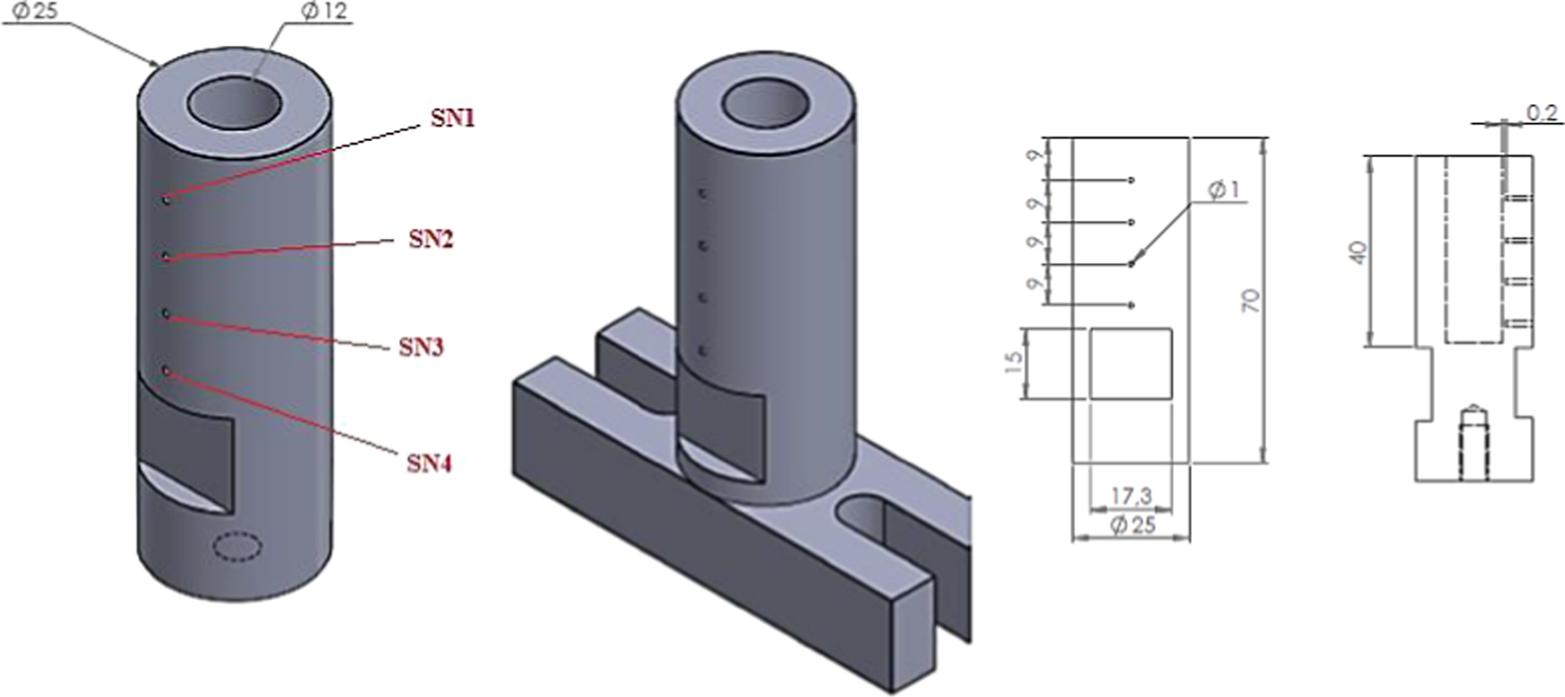
Technical drawing of temperature measurement samples.

**Figure 3:**
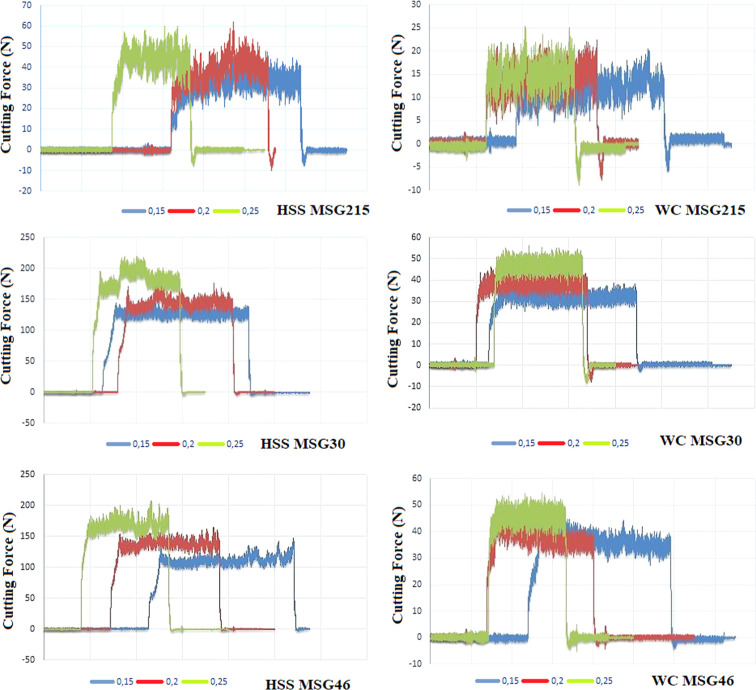
Force graphs for MSG215, MSG30, and MSG46 specialty graphite materials with (left) HSS drill bit and (right) WC-Co drill bits.

Six different hole drilling tests were conducted to investigate the temperatures. These tests were designed to represent the primary drilling conditions and have been replicated with one more test. Repeated tests showed similar regime and temperature changes, which proves fair repeatability. Specifically, by providing the highest and lowest cutting speeds and feed rates, a systematic comparison of temperature measurements was enabled under the most extreme conditions. Detailed information on the drilling parameters used in these tests is seen in Table 3. These parameters were taken from previous hole drilling studies. A technical drawing of the temperature measurement sample is represented in Figure 2.

**Table 3:** Test Parameters for Temperature Measurement

material	cutting speed (m/min)	feed rate (mm/rev)	cutting tool
MSG30	100	0.25	HSS
MSG30	100	0.15	HSS
MSG46	200	0.25	HSS
MSG215	200	0.15	HSS
MSG215	200	0.15	WC-Co
MSG30	200	0.25	WC-Co

Surface roughness values are generally required by end users in applications where graphite materials are used as precision surfaces or require assembly. The average surface roughness values were obtained from the inner surface of the samples obtained after thermal tests using a profilometer. Tool wear was examined by using an optical microscope after 81-hole drilling operations. The wear observed on carbide and high-speed steel tools was interpreted to provide insights into the tool life.

Finally, hole diameters were measured on machined samples to assess the hole quality and dimensional accuracy. Measurements were performed by using a ZEISS Contura CMM measuring device.

## Results and Discussion

### Force Results

Force results for drilling holes with three different graphite materials for two different cutting tools are shown in Table 4. Increases in cutting force were observed with high feed rate levels. The fluctuation of cutting force may be associated with microstructural heterogeneity and possible anisotropic characteristics of the graphite. Also, these fluctuations may also be influenced by local variations in grain distribution and porosity within the graphite structure. The maximum cutting force was measured at 176 newton. This level was reached via the HSS tool. The drilling speed was 50 m/min. The feed rate was 0.25 mm/rev. These values decreased nearly 4-fold with the carbide tool. The cutting force results with both the HSS and carbide tools increased as cutting speeds increased and the feed rate increased as well.

**Table 4:** Average Force Results for HSS and WC Drill Bit

cutting tool	particle size (mm)	cutting speed (m/min)	feed rate (mm/rev)	force (N)
HSS	1	50	0.15	30.81
HSS	1	75	0.2	39.98
HSS	1	100	0.25	48.37
HSS	0.1	50	0.2	155.07
HSS	0.1	75	0.25	172.34
HSS	0.1	100	0.15	124.93
HSS	0.09	50	0.25	175.74
HSS	0.09	75	0.15	105.7
HSS	0.09	100	0.2	135.15
WC-Co	1	100	0.15	12.15
WC-Co	1	150	0.2	14.75
WC-Co	1	200	0.25	17.36
WC-Co	0.1	100	0.2	39.7
WC-Co	0.1	150	0.25	49.34
WC-Co	0.1	200	0.15	33.6
WC-Co	0.09	100	0.25	47.76
WC-Co	0.09	150	0.15	37.21
WC-Co	0.09	200	0.2	41.92

The accumulation of dust during drilling of, especially, coarse-grained graphite can significantly influence cutting mechanics. Unlike ductile or metallic materials, graphite produces fine chips (similar to powder) that may accumulate within the hole and around the cutting edges. This accumulation can increase friction and promote redrilling of graphite powders, leading to higher thrust forces and increasing the force fluctuations.^15^ Therefore, efficient chip evacuation is essential to maintaining stable cutting conditions.

Under the same conditions, three times the cutting force was measured compared to that of the MSG215 material. Due to the isotropic nature of the material, stable cutting forces were obtained. The representation of all force diagrams is given in Figure 3. The measured force increased by approximately 1.65 times as both the feed rate and the cutting speed increased to the maximum. Compared with the MSG30 material, almost the same forces were measured. Analytical force models for traditional drilling procedures exist, but they typically assume homogeneous cutting conditions and were created for ductile metallic materials. Analytical force prediction is challenging for graphite materials due to the brittle fracture process, microstructural variability, and dust production during drilling. The experimental link between the thrust force and cutting parameters was thus represented by a polynomial regression model. Process optimization is made easier by this empirical technique, which enables the precise depiction of the observed patterns. The force equation obtained polynomially depending on drilling parameters is given in Table 5.

**Table 5:** Polynomial Force Equations

HSS	WC
*F* = 89.5 – 103*n* + 248*f* + 0.0499*Vc*	*F* = 26.9 – 29.6*n* + 105*f* + 0.0237*Vc*

Besides, the main effect plot of the two drill bits is given in Figure 4. An S/N analysis was performed, calculating that the lowest force would be the best under the best conditions. This graph revealed that for both drill bits, the particle size was the most effective parameter. The second most effective parameter was feed rate. Cutting speed has a limited effect for all conditions.

**Figure 4:**
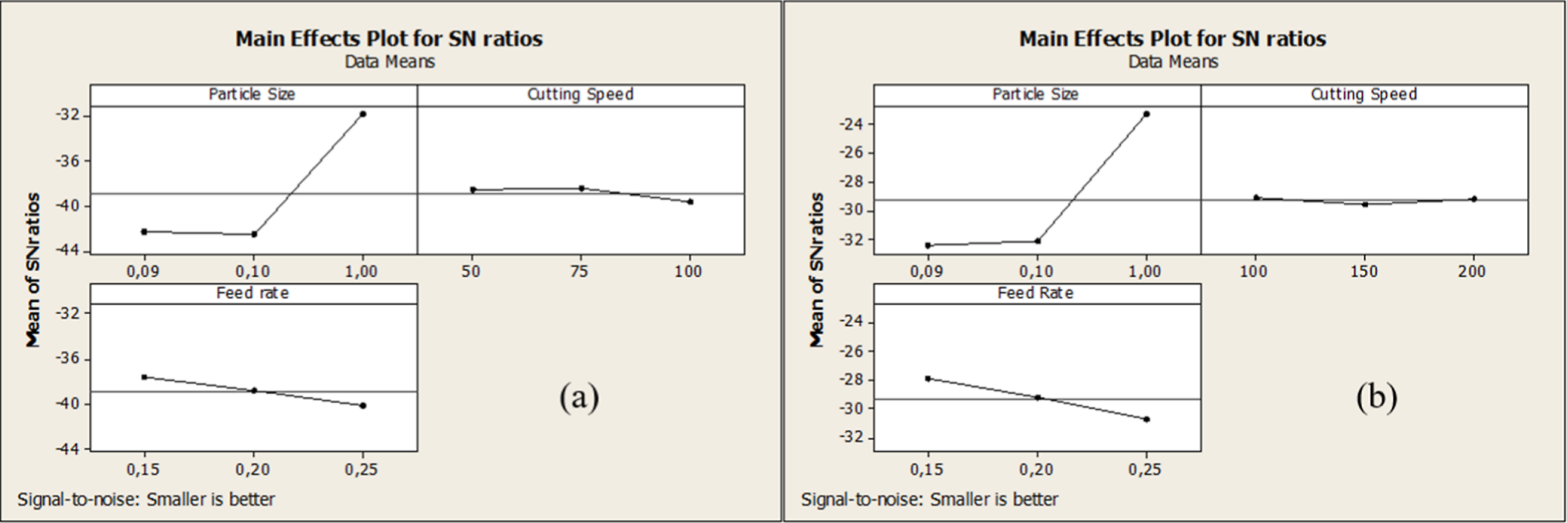
Main effect plot of (a) HSS drill bit and (b) WC-Co drill bit for force results.

Also, the interaction plot for all parameters is shown in Figure 5. A detailed examination of this graph reveals that for both HSS and carbide tools, the drilling speed is high at the lowest value when the maximum grain size is 0.09 mm for material, while the force output is high at the highest feed rate value. However, the highest force value at the 0.1 mm grain size, unlike the 0.09 mm grain size, occurs at the moderate drilling speed values and the highest feed rate values. A linear increase was observed in the coarse grain size.

**Figure 5:**
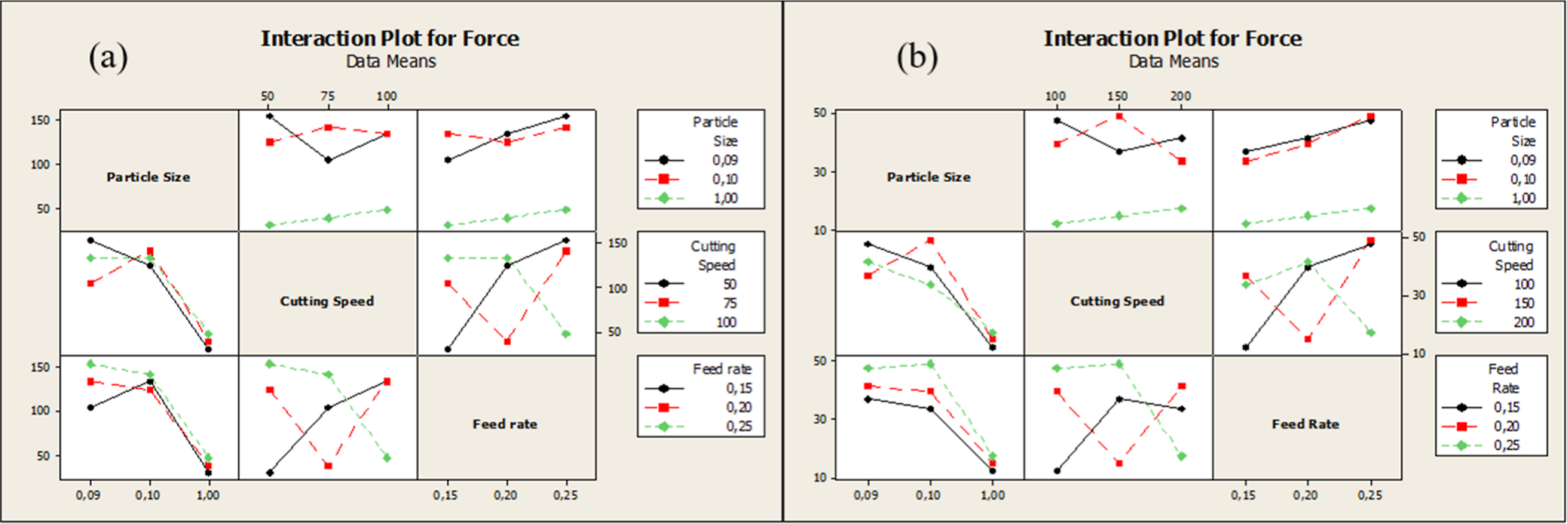
Interaction plot for force (a) HSS drill bit and (b) WC-Co drill bit.

If the material’s grain size is specifically considered, it is necessary to avoid low cutting speeds as the maximum grain size decreases. Especially when a low drilling speed value and high feed rate value cannot find the time and torque required for deformation of fine-grained graphite materials, it brings about high-strength concentrations. Even if you increase the feed rate and drilling speed, high-strength values could not reach. This demonstrates that especially when machining extremely fine graphite materials, high drilling speeds and feed values do not increase thrust forces. In short, the ANOVA results indicate that particle size is the dominant factor affecting thrust force, contributing to the largest variance in the model. Furthermore, the ANOVA results show that, accounting for about 91.9% of the total variation, particle size is the primary factor influencing the thrust force. On the other hand, cutting speed has a negligible impact (0.9%), whereas feed rate accounts for roughly 4.9%. The experimental model’s reliability is confirmed by its high coefficient of determination (*R* ^2^ = 97.75%) and low error contribution (2.2%). The ANOVA analysis for force results is represented in Table 6.

**Table 6:** ANOVA Analysis for Force Results

analysis of variance	degree of freedom	sum of squares	mean squares	*F* ratio (α = 5%)	*p*-value	contribution (%)
particle size	2	52402.4	26201.2	409.4	0.000	91.92
cutting speed	2	505.5	252.8	3.95	0.036	0.89
feed rate	2	2818.8	1409.4	22.02	0.000	4.94
error	20	1279.9	64.0			2.24
total	26	57006.7			R-Sq	97.75%

## Temperature Results

It is significant to investigate the workpiece temperature during drilling since it has a direct correlation with the parameters of the processed part. The generation of heat in drilling operations may have a detrimental impact on the workpiece, machine tool, and drill bit. This can cause manufacturing tolerances to deviate from the specified value, especially in precision operations. Dissipated heat is a phenomenon still being studied in machining operations. This section aims to understand the extent to which temperature increases occur in specialty graphites. Furthermore, it is estimated that heat will accumulate in the workpiece as a result of the high thermal conductivity of specialty graphite materials owing to their nature. Temperature measurement results are given in Figures 6, 7, and 8, respectively. All figures present the temperature increase measured at different depths during the drilling of MSG215, MSG40, and MSG30 graphite materials using WC-Co and HSS drill bits. In all cases, the temperature rapidly increased during the initial drilling stage and reached a peak value within approximately 8–10 s. In Figure 6, the temperature evolution during the drilling of MSG30 graphite uses HSS drills at a cutting speed of 100 m/min under two different feed rates (0.25 and 0.15 mm/rev). The results indicate that feed rate influences the duration of thermal exposure rather than significantly increasing the peak temperature during graphite drilling. In Figure 7, the maximum temperature observed for MSG215 was around 35 °C with HSS drills, whereas MSG46 exhibited slightly lower peak temperatures of approximately 26–27 °C with lower feed rates. In Figure 8, the maximum temperature observed for MSG215 was around 24–25 °C, whereas MSG30 exhibited slightly higher peak temperatures of approximately 26–27 °C. This difference can be attributed to the finer grain structure and higher mechanical strength of MSG30, which leads to increased cutting resistance and frictional heat generation. After reaching the peak, the temperature gradually decreased due to heat dissipation through the graphite material and the evacuation of graphite dust from the cutting zone.

**Figure 6:**
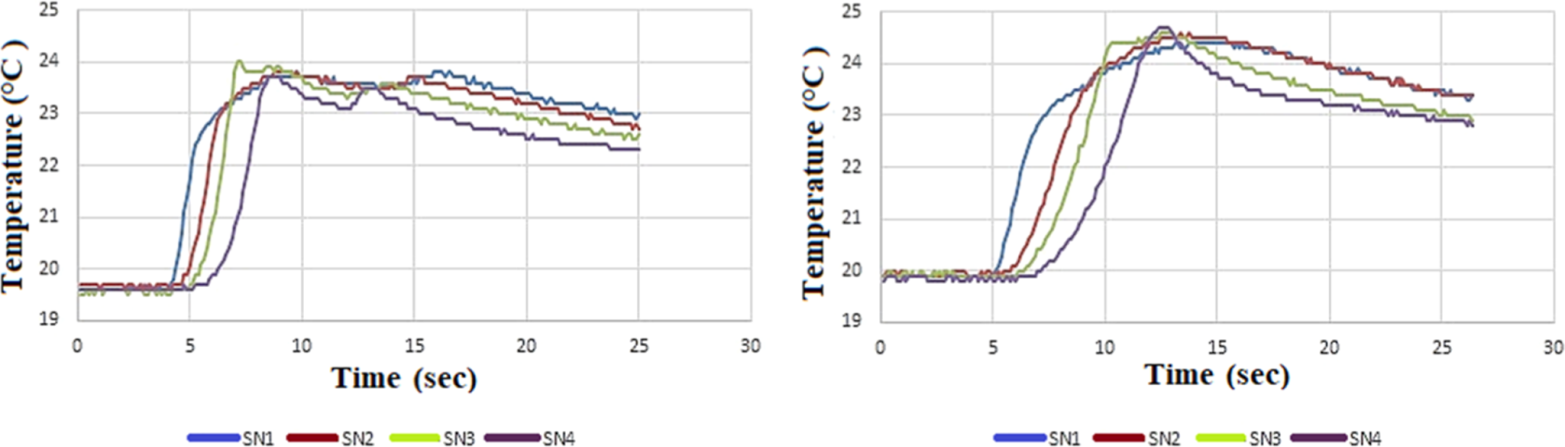
(a) MSG30-HSS material 100 m/min, 0.25 feed rate; (b) MSG30-HSS material 100 m/min, 0.15 feed rate temperature results.

**Figure 7:**
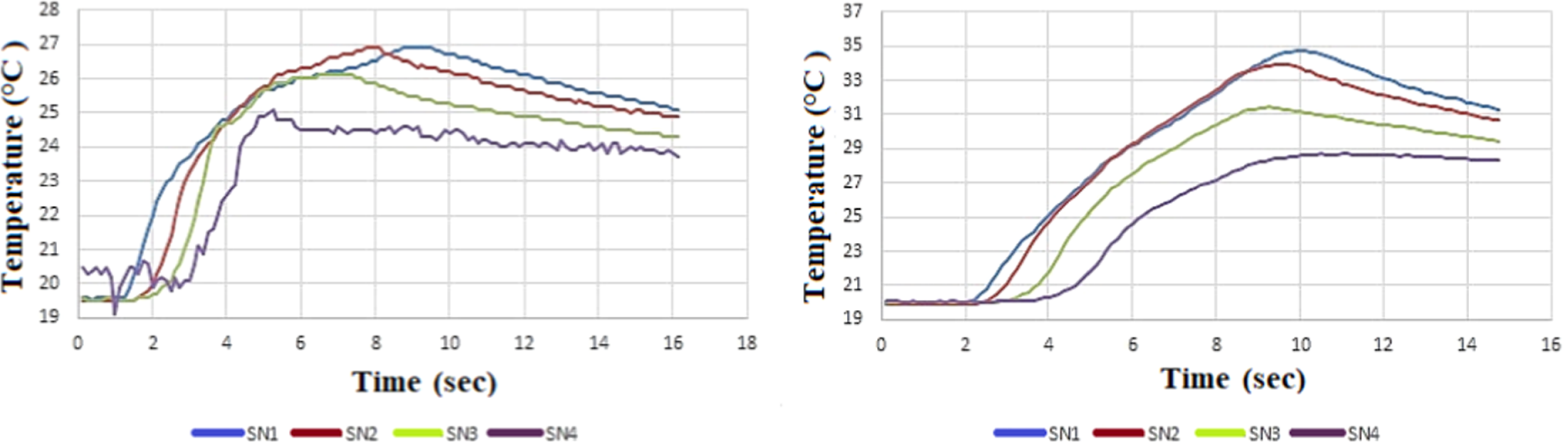
(a) MSG46-HSS material 200 m/min, 0.25 feed rate; (b) MSG215-HSS material 200 m/min, 0.15 feed rate temperature results.

**Figure 8:**
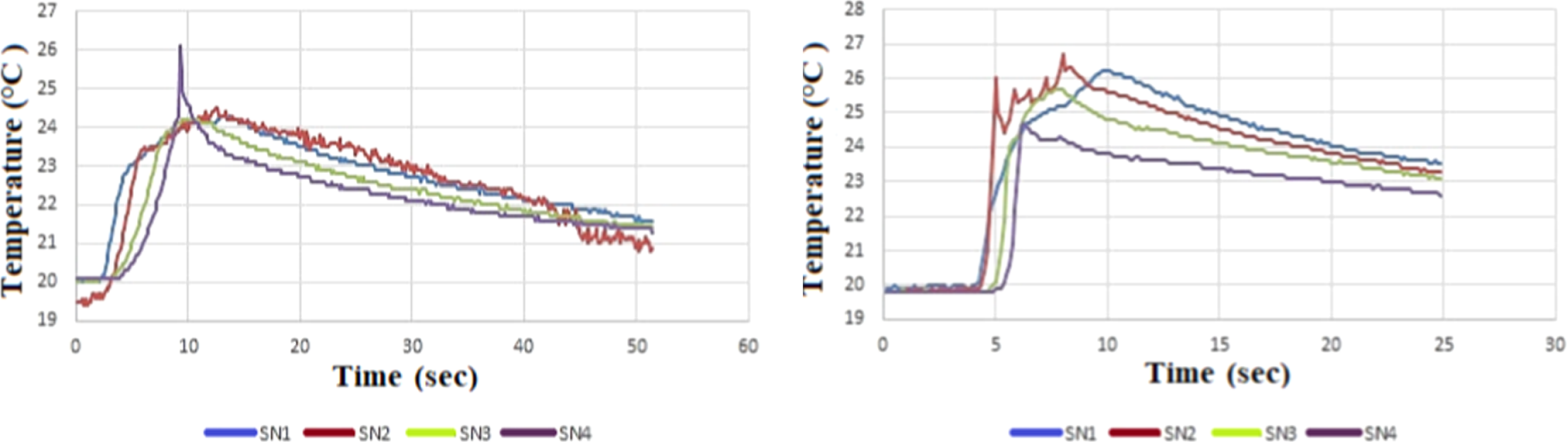
(a) MSG215-WC-Co material 200 m/min, 0.15 feed rate; (b) MSG30- WC-Co material 200 m/min, 0.15 feed rate temperature results.

This modest temperature increase observed during drilling can be attributed to the high thermal conductivity of graphite, which promotes rapid heat dissipation away from the cutting zone. In addition, heat is continuously removed by chip evacuation and tool conduction, limiting the thermal accumulation. Hence, the chip formation and processing characteristics of specialty graphites during hole drilling differ from those of metals. It has been observed that chips, especially dust, accumulate within the part, putting strain on the tool and increasing both the force and temperature. The porous microstructure of graphite may further facilitate heat diffusion, preventing localized temperature build-up. It can be apparently seen that the highest temperature change is at the lowest feed rate value. This is because there is not enough time for the temperature to increase at such a high rate of progression.

## Surface Roughness Results

The numbers in Table 7 represent the test numbers. The higher roughness values in parts 4 and 5 compared with the other samples stem from the average size of the parts. Due to the large grain size of the parts used here, differences were observed between the values obtained between the three measurements. The MSG46 material with the smallest grain size provided the best roughness results. Furthermore, it can be concluded that roughness values increase with an increased feed rate and that lower roughness values are achieved with carbide tools.

**Table 7:** Dimensional Accuracy and Surface Roughness of Graphite Materials

S no.	material	cutting speed (m/min)	feed rate (mm/rev)	cutting tool	tool diameter (mm)	hole diameter (CMM)	surface roughness (μm)
1	MSG30	100	0.25	HSS	11.992	11.9962	3.17 ± 0.2
2	MSG30	100	0.15	HSS	11.992	11.9918	2.53 ± 0.1
3	MSG46	200	0.25	HSS	11.992	11.9886	2.3 ± 0.3
4	MSG215	200	0.15	HSS	11.992	11.9997	3.44 ± 2.4
5	MSG215	200	0.15	WC-Co	11.997	12.0113	3.63 ± 0.65
6	MSG30	200	0.25	WC-Co	11.997	11.9908	2.76 ± 0.5

### Dimensional Accuracy Measurements

It is crucial that the hole produced during the drilling process be produced in one go with the desired dimensional accuracy and roundness. This section evaluates how these properties vary depending on the input parameters. The diameters (diameter deviation) and ovality (roundness deviation) of the holes obtained in the experimental studies were measured on a CMM machine.

### Tool Wear

The cutting forces obtained with the HSS tool were three to four times greater than those obtained with the carbide tool. After the examination of wear over the cutting tools in Figure 9, it is observed not as a continuous and homogeneous wear band, but rather as linear abrasion marks and edge deformation. Microchipping is observed in places on the cutting tool, which is typical for graphite. Parallel grooves observed on the flank face, which can be seen in Figure 9, indicate dominant abrasive wear caused by hard graphite particles. Furthermore, some progressive edge rounding suggests gradual material removal and loss of sharpness of the tools. Besides, localized microchipping at the cutting edge was observed. Therefore, although the conditions compared in the obtained micrographs were the same, the tool wear occurring with the HSS tool was 1.5–2 times greater than that of the carbide tool. Measured flank wear features ranged between approximately 50 and 130 μm, indicating localized wear progression rather than uniform wear land formation. Unlike ductile and metallic materials, graphite does not promote continuous wear. Instead, localized abrasion and microedge degradation can be seen in micrographs in Figure 9.

**Figure 9:**
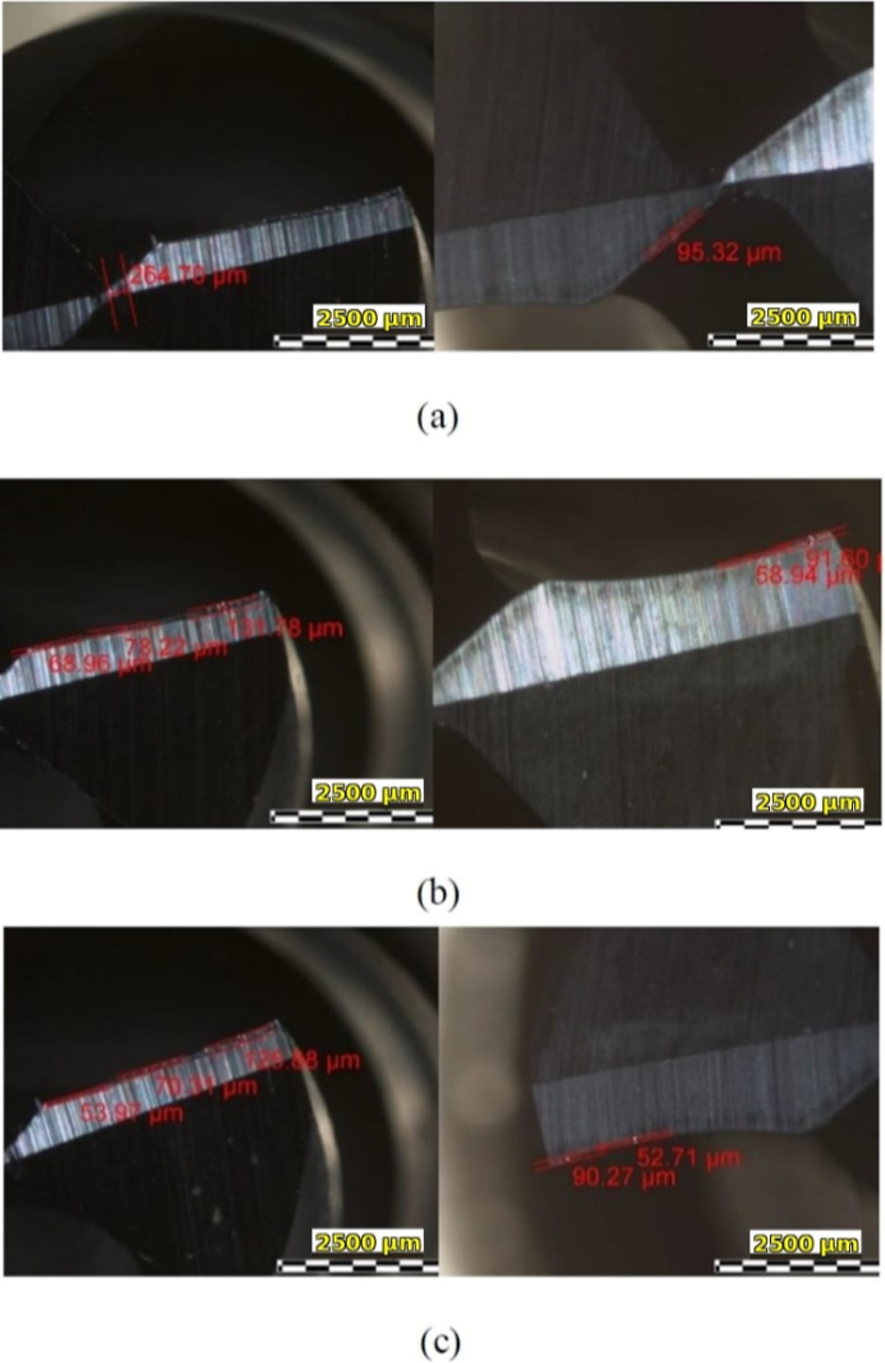
Micrographs of the tools after tests: (a) middle, (b) right, and (c) left.

## Conclusions

This study investigated the drilling performance of three specialty graphite materials with different grain sizes by using HSS and WC drills under varying cutting parameters. Based on the experimental findings, the following conclusions can be drawn:

(1) The primary material characteristic affecting drilling forces was found to be the graphite grain size. The coarse-grained graphite (MSG215) produced much lower thrust forces (48 N), whereas the extremely fine-grained graphite (MSG46) produced the highest thrust force (182 N).
(2) In the coarse-grained graphite (MSG215), force changes were more noticeable; at the same cutting conditions, variations of about 3–4 N were noted, suggesting the impact of microstructural heterogeneity.
(3) Because graphite has a strong thermal conductivity, the temperature rise during drilling was comparatively minimal. Under dry drilling conditions, the average workpiece temperature stayed about 35 °C, with a maximum temperature increase of about 15 °C.
(4) Tool wear and surface integrity were greatly impacted by grain size. Higher surface roughness was caused by coarse-grained graphite, and HSS drills were about twice as worn as WC drills.
(5) Stable drilling conditions are largely dependent on the effective removal of graphite dust. Chip evacuation, force fluctuations, and hole quality can all be improved by using air blowing, vacuum extraction, or peck drilling techniques.

Overall, the findings show that the microstructure of graphite, especially grain size, has a significant impact on drilling performance and should be carefully taken into account when choosing cutting parameters and tool materials for the precision machining of specialty graphite components.
